# The illusion of academic visibility: database selectivity, language hierarchies, and citation inequality in small-state legal scholarship

**DOI:** 10.3389/frma.2026.1938599

**Published:** 2026-10-23

**Authors:** Dejan Marolov, Elena Trajkovska

**Affiliations:** Faculty of Law, Goce Delcev University, Stip, North Macedonia

**Keywords:** academic visibility, bibliometrics, citation inequality, database selectivity, language bias, legal scholarship, scholarly communication, small-state academia

## Abstract

**Introduction:**

Academic visibility is increasingly shaped by inclusion in major bibliometric databases, yet the extent to which these systems accurately represent scholarly contributions from small academic communities remains contested. This study examines how database selectivity, language hierarchies, and citation inequality influence the visibility of legal scholarship in a small-state context.

**Methods:**

Using a comparative bibliometric analysis of Google Scholar, Scopus, and Web of Science, the study focuses on three leading law faculties in North Macedonia: Ss. Cyril and Methodius University in Skopje (UKIM), Goce Delcev University (UGD), and South East European University (SEEU).

**Results:**

The findings reveal a layered structure of academic visibility shaped by differences in database coverage and indexing practices. While Google Scholar captures a broad spectrum of scholarly output, Scopus and Web of Science provide increasingly selective representations, resulting in substantially lower levels of measurable research activity. Across the analyzed institutions, citation impact is unevenly distributed, with a small number of researchers accounting for a disproportionate share of total citations, although the precision of inequality estimates varies across institution-database combinations. Publication language emerges as an important factor associated with differences in citation visibility. English-language publications account for the dominant share of citations across the analyzed databases, whereas Macedonian- and Albanian-language outputs show substantially lower citation visibility despite their presence in overall publication volume.

**Discussion:**

The findings suggest that international visibility is associated not only with research productivity, but also with linguistic and structural characteristics embedded within contemporary systems of scholarly communication. Academic visibility in small-state contexts is socially and institutionally constructed through the interaction of database inclusion, language dominance, and cumulative citation dynamics. The study contributes to debates on research evaluation and academic publishing by demonstrating how bibliometric systems may produce partial representations of scholarly activity, particularly in disciplines characterized by strong local and national orientations. The findings highlight the need for more context-sensitive approaches to assessing research impact in peripheral academic systems.

## Introduction

Academic visibility has become a central feature of contemporary scholarly communication. Inclusion in major bibliometric databases such as Google Scholar, Scopus, and Web of Science increasingly influences how research is discovered, evaluated, cited, and recognized within the global academic community. These databases function not only as repositories of scholarly output but also as infrastructures of academic visibility, shaping perceptions of research impact and scholarly influence (Harzing and Alakangas, 2016; Mongeon and Paul-Hus, 2016).

Despite their widespread use, bibliometric databases differ considerably in their coverage, indexing practices, and selection criteria. Google Scholar provides broad and inclusive coverage of scholarly materials, whereas Scopus and Web of Science apply more selective standards focused primarily on internationally indexed publications (Harzing and Alakangas, 2016; Mongeon and Paul-Hus, 2016). As a result, different databases often produce different representations of the same academic community. This raises important questions regarding the extent to which academic visibility reflects actual scholarly activity or, alternatively, the structural characteristics of the databases through which that activity is measured.

These issues are particularly relevant in smaller academic systems and in disciplines such as law. Legal scholarship is often closely connected to national legislation, judicial practice, and domestic institutional frameworks (Kulczycki et al., 2020). Consequently, a substantial proportion of legal research is published in local languages and directed toward national audiences. While such publications may play an important role within domestic scholarly and professional communities, they frequently remain underrepresented in international bibliometric databases and attract limited citation visibility beyond their immediate context (Van Leeuwen et al., 2001; Kulczycki et al., 2020).

The relationship between database inclusion, language of publication, and citation performance has attracted growing scholarly attention. Previous studies have documented substantial differences in database coverage (Harzing and Alakangas, 2016; Mongeon and Paul-Hus, 2016), the dominance of English in international scholarly communication (Van Leeuwen et al., 2001; Meneghini et al., 2008; Kulczycki et al., 2020), and the unequal distribution of citations among researchers (Merton, 1968; Larivière et al., 2009; Waltman, 2016). However, these dimensions are often examined separately, while their combined effects on academic visibility remain insufficiently explored. This gap is particularly evident in small-state academic systems, where local publication practices, linguistic diversity, and limited international visibility intersect in distinctive ways.

This study examines how academic visibility is constructed and distributed within legal academia in North Macedonia. Focusing on three leading law faculties—Faculty of Law “Iustinianus Primus” at Ss. Cyril and Methodius University in Skopje (UKIM), Faculty of Law at Goce Delcev University (UGD), and Faculty of Law at South East European University (SEEU)—the research compares patterns of database coverage, citation impact, and language distribution across Google Scholar, Scopus, and Web of Science.

Rather than treating visibility as a neutral reflection of research quality, the paper approaches it as a structurally conditioned phenomenon shaped by the interaction of database inclusion, language hierarchies, and cumulative citation dynamics (Merton, 1968; Hicks et al., 2015; Mongeon and Paul-Hus, 2016). Through a comparative bibliometric analysis, the study explores how different indexing systems construct distinct representations of scholarly activity and how these representations influence the recognition of research produced in peripheral academic environments.

To address this gap, the study examines academic visibility through a comparative bibliometric analysis of Google Scholar, Scopus, and Web of Science. Particular attention is given to database coverage, citation impact, language distribution, and patterns of citation concentration among researchers. By comparing three leading law faculties in North Macedonia, the study seeks to identify the mechanisms through which academic visibility is produced, measured, and unevenly distributed within a small-state academic system.

The study is guided by the following research questions:

RQ1: how do Google Scholar, Scopus, and Web of Science differ in their coverage of legal academic staff in North Macedonia?
RQ2: to what extent does research visibility differ across databases in terms of measurable citation impact?
RQ3: what is the distribution of citation impact among researchers within each institution?
RQ4: how does citation performance vary across publication languages and databases?
RQ5: how do differences in database coverage and indexing scope shape the representation of legal scholars' research output?

By focusing on legal scholarship within a small-state context, the article contributes to ongoing debates on scholarly communication, research evaluation, and academic publishing. It argues that academic visibility is not determined solely by research productivity, but also by the infrastructures, through which knowledge is indexed, disseminated, and recognized. Understanding these mechanisms is essential for developing more balanced and context-sensitive approaches to assessing scholarly impact in diverse academic environments.

### Theoretical framework and literature review

The question of academic visibility has become increasingly central in contemporary research evaluation, particularly within the context of globalized knowledge production. Bibliometric indicators such as citation counts, h-index, and database inclusion are widely used as proxies for academic impact. However, a growing body of literature suggests that these indicators do not merely reflect research quality, but are shaped by structural inequalities embedded within the global academic system. Rather than functioning as neutral measurement tools, bibliometric systems play an important role in shaping academic visibility and recognition (Hicks et al., 2015).

Within this framework, three key structural dimensions are central to understanding patterns of academic visibility: database selectivity, language hierarchies, and citation inequality.

First, a substantial body of research highlights the role of database bias in shaping academic visibility. Major indexing platforms such as Web of Science and Scopus apply selective inclusion criteria that can result in the underrepresentation of research produced in smaller or peripheral academic systems (Mongeon and Paul-Hus, 2016; Singh et al., 2021). More recent comparative evidence confirms substantial differences in citation coverage across bibliometric databases, with Google Scholar providing considerably broader coverage than Scopus and Web of Science across multiple disciplines (Martín-Martín et al., 2021). These differences are particularly relevant for legal scholarship, where traditional citation databases such as Web of Science index a comparatively limited number of law journals (Beatty, 2020). Moreover, research on legal journal evaluation indicates that a substantial proportion of journals regarded by legal scholars as internationally leading are not indexed in Web of Science, while legal publishing and citation patterns retain significant regional characteristics (Hojnik, 2021). Consequently, database inclusion should not be treated as a neutral proxy for the totality or quality of scholarly activity, particularly in disciplines with strong national and regional publication traditions. Recent research further highlights that database inclusion is shaped by evolving selection criteria and changing definitions of what constitutes valuable scientific output, particularly within major indexing systems such as Web of Science (Vanderstraeten, 2025).

Second, publication language is closely associated with patterns of academic visibility and scholarly recognition. English has become the dominant language of international scholarly communication, while research published in other languages often remains more closely connected to national or regional scholarly communities (Van Leeuwen et al., 2001; Meneghini et al., 2008). This linguistic imbalance is particularly relevant to legal scholarship. Ammann (2022) identifies the dominance of English as one of the clearest manifestations of language bias in international legal scholarship and argues that such bias can shape the accessibility, dissemination, and recognition of legal knowledge. While the use of English as a common scholarly language facilitates communication with wider international audiences, it may also disadvantage scholarship produced in other languages and contribute to unequal patterns of academic visibility (Ammann, 2022). This creates an important tension between international visibility and the continued relevance of scholarship produced for national and regional academic and professional communities.

Multilingual scholarship remains particularly important in the social sciences and humanities, where research is frequently oriented toward national and local audiences. Evidence from European academic systems demonstrates substantial variation in publication-language patterns across countries and disciplines, with local-language publishing remaining especially prominent in fields such as law, history, and the humanities (Kulczycki et al., 2020). This suggests that the limited representation of local-language scholarship in international bibliometric databases should not be interpreted as evidence of limited scholarly relevance, since such publications may serve important disciplinary, professional, and societal functions within their national contexts. Recent empirical research on legal scholarship has also employed citation-based approaches to examine patterns of scholarly output and impact within specific institutional and disciplinary contexts (Sheehy and Dumay, 2021).

Third, citation distribution is characterized by strong inequality, commonly explained through cumulative advantage mechanisms such as the Matthew effect, whereby already highly cited researchers tend to attract disproportionately more citations over time (Merton, 1968; Larivière et al., 2009). Bibliometric studies consistently show that citation counts are highly skewed, with a small number of researchers or publications accounting for a disproportionate share of total citations (Larivière et al., 2009; Waltman, 2016). This concentration is further reinforced by database inclusion, institutional prestige, and prior visibility, leading to a self-reinforcing cycle of academic recognition. Consequently, citation-based indicators tend to amplify existing inequalities rather than provide a fully neutral reflection of research impact (Merton, 1968; Hicks et al., 2015).

Despite these advances, the existing literature exhibits two important limitations. First, most studies focus on large or globally dominant academic systems, while smaller and less internationally integrated contexts remain underexplored. Second, limited attention has been given to the interaction between database selectivity, language of publication, and citation inequality within specific disciplinary fields such as law, where publication practices differ significantly from those in the natural sciences. Recent developments in responsible research assessment further challenge the use of bibliometric indicators as stand-alone proxies for research quality. The Leiden Manifesto emphasizes that quantitative indicators should support, rather than replace, qualitative expert assessment and that evaluation should account for disciplinary differences and the value of locally relevant research (Hicks et al., 2015). These principles have been further developed through the Agreement on Reforming Research Assessment, which advocates recognition of diverse research outputs, activities, and contributions and the responsible use of quantitative indicators alongside qualitative judgement (Agreement on Reforming Research Assessment, 2022). At the institutional level, the responsible metrics framework similarly emphasizes disciplinary context, data quality, transparency, diversity, and awareness of potential unintended consequences in research assessment (Wilsdon et al., 2015). Taken together, these approaches caution against treating citation counts, database inclusion, or individual bibliometric indicators as stand-alone measures of research quality.

For the purposes of this study, academic visibility refers to the extent to which researchers and their scholarly outputs are represented and bibliometrically observable within academic databases. It is operationalized through database representation, publication output, and measurable citation indicators. Database selectivity refers to differences in the scope, inclusion criteria, and indexing practices through which bibliometric databases determine which scholarly outputs and publication venues are represented; in this study, it is examined comparatively through differences in researcher coverage and indexed publication output across Google Scholar, Scopus, and Web of Science. Language hierarchy refers to the unequal patterns of bibliometric visibility associated with different publication languages, particularly the dominant position of English within international scholarly communication; it is examined through the distribution of publications and citations across English, Macedonian, Albanian, and other languages. Citation inequality refers to the uneven distribution of citations across researchers and is measured using median citation counts, interquartile ranges (IQR), zero-citation proportions, and Gini coefficients.

Importantly, database selectivity, language hierarchy, and citation inequality are not conceptualized here as exhaustive constituent components of academic visibility. Rather, they are treated as analytically distinct dimensions through which bibliometric visibility can vary across databases, publication languages, institutions, and researchers. The study therefore does not seek to construct a composite measure of academic visibility or to explain individual scholarly capability or research quality. Instead, it examines how observable scholarly activity is represented within different bibliometric systems and how that representation varies according to database coverage, publication language, and the distribution of citations. Researcher-level characteristics such as age, career stage, workload, institutional responsibilities, or other individual attributes may also be associated with citation performance, but examining their explanatory effects falls outside the comparative bibliometric scope of the present study. Career age was not independently measured in the present dataset. Although the year of first indexed publication was recorded where available, this indicator was not used as a proxy for career age because it is database-dependent and may not correspond to the actual beginning of an academic career.

Building on these gaps, this study conceptualizes academic visibility as a structurally conditioned and multi-layered phenomenon that cannot be fully captured through conventional bibliometric indicators alone. By providing a comparative bibliometric analysis of legal academia in North Macedonia, the study examines how patterns of academic visibility vary in relation to database inclusion, language of publication, and citation distribution within a small-state academic system. In doing so, it contributes to a more nuanced understanding of how academic impact is produced, measured, and unevenly distributed in peripheral academic contexts.

## Methodology

This study provides a systematic comparative bibliometric analysis of legal scholarship in a small-state context and adopts a comparative research design to examine academic visibility, citation impact, and structural inequalities within legal academia in North Macedonia.

The analysis focuses on three major law faculties: the Faculty of Law “Iustinianus Primus” at Ss. Cyril and Methodius University in Skopje (UKIM), the Faculty of Law at Goce Delcev University (UGD), and the Faculty of Law at South East European University (SEEU). These institutions were selected due to their central role within the national academic system and their representation in international rankings. According to the SCImago Institutions Rankings (SCImago Research Group, 2025), the most recent data available at the time of analysis, these institutions represent the only higher education institutions from the country included in this global ranking system.

The study includes the full population of academic staff affiliated with the selected law faculties, thereby avoiding sampling within the selected institutions and ensuring comprehensive coverage of the defined study population. Although limited in absolute size, this population captures the core of legal academia in the national context and allows for a systematic analysis of database coverage, citation patterns, and language-related dynamics. Academic staff included in the study were identified from the official staff lists published on the websites of the three law faculties. These official institutional webpages served as the reference source for defining the study population at the time of data collection. The analysis included all academic staff listed on the respective faculty websites during the data collection period. Because institutional websites are periodically updated, the staff lists used in this study reflects the composition of academic staff as available at the time of data collection.

The academic staff population also differed in terms of academic rank and employment status. At UGD, the 21 staff members comprised 14 full professors (66.7%), four associate professors (19.0%), one assistant professor (4.8%), and two external teaching staff members (9.5%). At UKIM, the 69 staff members comprised 60 full professors (87.0%), seven associate professors (10.1%), and two assistant professors (2.9%). At SEEU, the 21 staff members comprised 11 full professors (52.4%), six associate professors (28.6%), and four assistant professors (19.0%). These differences in staff composition are relevant when interpreting cross-institutional citation patterns, since academic rank may be associated with career length and accumulated publication and citation opportunities.

The empirical analysis is based on data extracted from three major academic databases—Google Scholar, Scopus, and Web of Science—which represent different levels of inclusiveness and selectivity. Google Scholar is treated as a broad-coverage database, while Scopus and Web of Science represent more selective indexing systems focused on internationally recognized publications. This enables a layered assessment of academic visibility across platforms.

The unit of analysis is the individual academic staff member affiliated with the selected faculties. The dataset includes both researchers with active profiles and those without representation in specific databases, allowing for a distinction between nominal visibility (presence in a database) and effective visibility (availability of measurable bibliometric indicators such as publications, citations, and index values).

For the purposes of the analysis, database representation and availability of citation data were treated as distinct concepts. Database representation refers to the identification of a researcher or indexed scholarly output associated with that researcher within a given bibliometric database. Availability of citation data refers more narrowly to cases in which researcher-level citation information could be retrieved and verified for inclusion in the citation-distribution analysis. A researcher could therefore be represented in a database without necessarily having citation data available for the researcher-level distributional analysis. These distinctions are reported separately where relevant.

Database representation and the availability of researcher-level citation data were treated as distinct concepts. For Scopus, a researcher was considered represented when an identifiable Scopus author profile was found. In a limited number of cases, an author profile could be identified, but the profile could not be opened or its researcher-level citation data could not be retrieved at the time of data collection; these cases were classified as “inaccessible profiles.” Such researchers were included in the database-representation figures reported in Table 1 but excluded from the profile-based citation-distribution analysis in Table 2. For Web of Science, representation was operationalized through the identification of at least one indexed publication attributable to the researcher. Accordingly, representation figures across databases reflect database-specific forms of bibliometric presence and should not be interpreted as strictly equivalent measures of author-profile availability.

**Table 1:** Comparative bibliometric indicators across Google Scholar, Scopus, and Web of Science for the three selected law faculties in North Macedonia.

Institution	Staff	Database	Coverage *n*/*N* (%)	Publications	Citations	Citations per publication
UKIM	69	Google Scholar	37/69 (53.6%)	1,665	3,244	1.95
UKIM	69	Scopus	45/69 (65.2%)	142	277	1.95
UKIM	69	Web of Science	47/69 (68.1%)	155	531	3.43
UGD	21	Google Scholar	21/21 (100.0%)	1,518	912	0.60
UGD	21	Scopus	18/21 (85.7%)	95	119	1.25
UGD	21	Web of Science	14/21 (66.7%)	53	91	1.72
SEEU	21	Google Scholar	13/21 (61.9%)	541	922	1.70
SEEU	21	Scopus	15/21 (71.4%)	86	150	1.74
SEEU	21	Web of Science	18/21 (85.7%)	92	229	2.49

Database coverage refers to the number and percentage of academic staff represented in each database (*n*/*N*). Representation is database-specific: in Scopus, it refers to researchers for whom an identifiable author profile was found, including profiles for which researcher-level citation data could not be retrieved at the time of data collection; in Web of Science, it refers to researchers with at least one identifiable indexed publication. Coverage figures therefore indicate database representation and should not be interpreted as strictly equivalent measures of researcher-level citation-data availability across databases.

**Table 2:** Citation inequality indicators across institutions and bibliometric databases.

Faculty	Database	*N*	Median citations	IQR	Researchers with zero citations *n* (%)	Gini coefficient	95% bootstrap CI
UKIM	Google Scholar	37	27	17–87	1 (2.7%)	0.660	0.508–0.734
UKIM	Scopus	27	4	1–9.5	5 (18.5%)	0.713	0.537–0.783
UKIM	Web of Science	47	1	0–6	16 (34.0%)	0.840	0.708–0.877
UGD	Google Scholar	21	20	10–66	0 (0.0%)	0.563	0.404–0.653
UGD	Scopus	14	6	1–9.75	4 (28.6%)	0.571	0.317–0.713
UGD	Web of Science	14	0	0–7.25	8 (57.1%)	0.745	0.547–0.872
SEEU	Google Scholar	13	44	29–59	0 (0.0%)	0.575	0.241–0.680
SEEU	Scopus	14	2.5	0.5–4.75	4 (28.6%)	0.807	0.306–0.859
SEEU	Web of Science	18	1	0–2.75	6 (33.3%)	0.851	0.607–0.896

*N* refers to researchers with accessible citation data. Researchers without a database profile and those with inaccessible profiles were excluded from citation-distribution calculations. Duplicate profiles were not counted as separate researchers. Researchers with accessible profiles and zero citations were retained in the analysis. IQR represents the 25th−75th percentile range. Gini coefficients are reported with 95% non-parametric bootstrap confidence intervals based on 10,000 resamples. Wider confidence intervals, particularly in institution–database combinations with small *N*, indicate greater uncertainty in the estimated degree of citation inequality.

For each academic staff member identified from the official faculty websites, bibliometric profiles were searched independently in Google Scholar, Scopus, and Web of Science using the researcher's full name. Profile identification was verified primarily through institutional affiliation, whenever available, and by cross-checking publicly available bibliographic information. In cases where institutional affiliation was not explicitly indicated, additional publicly available information, including the researcher's field of expertise and publication profile, was used to determine whether the retrieved profile corresponded to the respective academic staff member. Where profile identification remained ambiguous, additional publicly available bibliographic information was consulted before the profile was included in the dataset. In a small number of cases, duplicate author profiles were identified. Where this occurred, profile information was reviewed and the profile corresponding to the researcher's institutional affiliation and publication profile was used for data extraction. To minimize identification errors arising from different spelling conventions, researchers were searched using multiple name variants where necessary, including both Latin and Cyrillic transliterations and alternative spellings appearing across bibliometric databases.

The analysis was based on all publications recorded in the identified author profiles within Google Scholar, Scopus, and Web of Science at the time of data collection. For each researcher, bibliometric indicators, including the total number of publications, total citation count, h-index, i10-index (where available), year of first indexed publication, and language of publication, were extracted as reported by each database, thereby reflecting the coverage and indexing practices specific to each bibliometric source. Citation counts were further disaggregated by language to assess the relationship between publication language and citation impact.

Publications were classified according to the language of the full publication rather than the language of the title or abstract. Language was initially identified from the publication title and accompanying bibliographic information. Where the language could not be determined with certainty, the publication itself was consulted to verify the language of the full text. Publications were subsequently classified into four categories: English, Macedonian, Albanian, and Other. Because publications in languages other than English, Macedonian, and Albanian were aggregated into a heterogeneous “Other languages” category, they were retained in the overall publication counts and reported descriptively where relevant, but were not treated as a separate homogeneous language group in the formal cross-language comparison. The “Other languages” category includes publications in languages other than English, Macedonian, and Albanian, primarily Serbian, as well as Russian, German, Slovenian, and other languages identified during data collection. These languages were aggregated into a single category in the original dataset and therefore could not be reliably disaggregated retrospectively.

The analytical approach combines descriptive and comparative methods. Descriptive statistics were used to calculate aggregate indicators at the institutional level, including total publications, total citations, average citations per researcher, and citations per publication. Comparative analysis across databases evaluated differences in coverage, indexing practices, and measurable research impact. To assess citation inequality more systematically, the analysis calculated the median citation count, interquartile range (IQR), proportion of researchers with zero citations, and Gini coefficient for each institution and bibliometric database. The IQR represents the range between the 25th and 75th percentiles, while the Gini coefficient was used as a measure of citation concentration, with higher values indicating greater inequality in the distribution of citations. These calculations included researchers with accessible citation data, including those with confirmed zero citations, while researchers without a database profile, those with inaccessible profiles, and duplicate profiles were excluded from the citation-distribution calculations.

Given the relatively small number of researchers with accessible citation data in some institution–database combinations, 95% bootstrap confidence intervals were calculated for the Gini coefficients to assess the precision of the inequality estimates. Confidence intervals were estimated using 10,000 non-parametric bootstrap resamples with replacement from the observed researcher-level citation distributions, using the 2.5th and 97.5th percentiles of the bootstrap distribution.

To assess whether differences in database coverage affected the observed citation-distribution patterns, an additional full-population sensitivity analysis was conducted. In this analysis, the complete academic staff population of each institution was retained (UGD, *N* = 21; UKIM, *N* = 69; SEEU, *N* = 21). Researchers for whom no profile or no accessible citation count was available in a given database were assigned zero bibliometrically observable citations for the purposes of this sensitivity test. This coding does not imply that these researchers had no citations in an absolute sense; rather, it operationalizes their lack of observable citation impact within the database under examination. Median citations, interquartile ranges, zero-observation shares, and Gini coefficients were then recalculated using the full institutional denominator. The original profile-based analysis was retained as the primary analysis, while the full-population specification was used to test the robustness of cross-database differences to variations in researcher coverage.

Key concepts were operationalized as follows: academic visibility refers to database presence combined with measurable bibliometric indicators; citation impact is measured through total citation counts and citations per publication; database coverage refers to the proportion of academic staff represented within each database; publication volume refers to the total number of publications; and language of publication is categorized into English, Macedonian, Albanian, and Other.

The study adopts a descriptive and comparative bibliometric design. Its purpose is not to estimate causal relationships or individual-level determinants of citation performance, but to compare patterns of researcher representation, publication output, citation visibility, and citation distribution across bibliometric databases and institutions.

These procedures were applied consistently across all three institutions and bibliometric databases, providing a standardized protocol for identifying researchers, extracting bibliometric indicators, classifying publication language, and calculating the reported measures. This procedure enables the analysis to be replicated using the same institutional staff lists, database sources, identification criteria, and analytical definitions.

The analysis is based on a cross-sectional dataset reflecting academic profiles at a specific point in time. Data collection was conducted between 1 February 2026 and 15 April 2026, and the findings should therefore be interpreted as a temporal snapshot of academic visibility. Differences in database coverage, indexing criteria, citation windows, and author-profile systems limit direct comparability across platforms. Citation counts should therefore be interpreted within the specific coverage and temporal scope of each database rather than as directly equivalent measures of research impact. In addition, because Google Scholar profiles are partly user-managed and may include automatically assigned publications, occasional inaccuracies in publication attribution cannot be entirely excluded. These limitations reflect inherent characteristics of the underlying bibliometric databases and the use of publicly available profile data. Given the descriptive and cross-sectional design of the study, observed differences in citation impact across publication languages are interpreted as associations and should not be understood as evidence of a causal effect of language on citation performance.

### Faculty of Law, Goce Delcev University (Google Scholar Analysis)

The analysis of Google Scholar data for the Faculty of Law at Goce Delcev University (UGD) includes a total of 21 academic staff members, all of whom maintain active Google Scholar profiles, indicating full database coverage within this platform.

In terms of research output, a total of 1,518 publications were identified. The distribution of publications by language reveals a relatively balanced structure, with 896 publications (59.0%) in English, 602 publications (39.7%) in Macedonian, and 20 publications (1.3%) in other languages.

Despite this relatively balanced distribution of research output, citation patterns reveal a pronounced asymmetry. English-language publications account for 845 citations (92.7%), while Macedonian-language publications account for 66 citations (7.2%). Publications in other languages account for only 1 citation (0.1%).

When measured in terms of citations per publication, the difference remains substantial. English-language publications receive approximately 0.94 citations per publication, compared with approximately 0.11 citations per publication for Macedonian-language outputs. Citation distribution across individual researchers further demonstrates a high degree of inequality. The most cited researcher records 238 citations, while several researchers have only single-digit citation counts. This pattern indicates that a disproportionate share of total citations is concentrated among a small number of individuals, while the majority of researchers exhibit relatively low citation impact. This dynamic is consistent with cumulative advantage mechanisms and suggests that overall academic visibility is driven by a limited subset of highly visible scholars.

Taken together, these findings reveal a clear divergence between the distribution of research output and citation impact. While publication activity appears relatively balanced across languages, citation impact is overwhelmingly concentrated in English-language outputs. The observed pattern indicates a strong association between publication language and citation visibility within the analyzed dataset, without implying a causal effect of language on citation performance.

### Faculty of Law, Goce Delcev University (Scopus Coverage and Research Visibility)

The analysis based on Scopus data provides a more selective perspective on indexed research output and citation impact. Out of a total of 21 academic staff members, 18 (85.7%) maintain identifiable Scopus profiles, while three (14.3%) are not represented in this database. However, profile presence does not necessarily correspond to effective research visibility. Several profiles display low or zero citation counts, indicating uneven levels of measurable impact within the indexed system. Researcher-level citation data were accessible for 14 of these 18 identifiable profiles; for the remaining four profiles, the required researcher-level citation data could not be reliably retrieved during the data-collection period.

In terms of research output, a total of 95 Scopus-indexed documents were identified across the analyzed sample, generating 119 citations. All identified publications are in English, reflecting strong alignment with international publication standards and indexing criteria.

With regard to research impact, the average h-index is 1.29, indicating a modest but measurable level of citation performance within the Scopus-indexed environment. Measured in terms of citations per publication, the average reaches approximately 1.25 citations per publication.

In comparison to Google Scholar data, which reflects a substantially larger volume of publications, the Scopus dataset reveals a substantially lower number of indexed outputs. However, these publications are concentrated in internationally visible and peer-reviewed sources, highlighting the trade-off between inclusiveness and selectivity across databases.

Citation patterns across individual researchers reveal a clear degree of concentration. A limited number of profiles account for a disproportionately large share of citations, while several researchers exhibit low or zero citation values, indicating uneven research visibility within the indexed environment. This concentration is particularly pronounced at the top of the distribution, where a single researcher accounts for more than one-third of total citations (43 out of 119). Notably, the same researcher is also the most cited within the Google Scholar dataset, indicating a consistent pattern of academic visibility across different indexing platforms.

Taken together, these findings indicate that although Scopus provides a more selective dataset, research visibility remains uneven and concentrated among a limited number of individuals. This further supports the argument that database inclusion alone does not guarantee broad or evenly distributed academic impact.

### Faculty of Law, Goce Delcev University (Web of Science Coverage and Research Activity)

The Web of Science (WoS) analysis of the Faculty of Law at Goce Delcev University reveals substantially lower coverage compared to Google Scholar. Out of a total of 21 academic staff members, 14 (66.7%) are represented through publications indexed in the WoS database, while 7 (33.3%) have no indexed publications.

In terms of research output, a total of 53 WoS-indexed publications were identified across the analyzed sample, all of which are published in English. No publications in Macedonian or Albanian were recorded in the analyzed WoS dataset. With regard to research impact, the total number of citations amounts to 91, corresponding to an average of approximately 6.5 citations per researcher with WoS-indexed publications, indicating a moderate level of citation performance within the indexed dataset.

In comparison to Google Scholar, differences in coverage and citation visibility become particularly pronounced. While Google Scholar includes all academic staff and captures a substantially larger volume of publications and citations, Web of Science reflects a far more selective indexing system, resulting in reduced representation of overall academic output.

Citation patterns across individual researchers reveal a clear degree of concentration. A limited number of researchers account for a disproportionately large share of citations, while several researchers record low or zero citation values, indicating uneven research visibility within the WoS-indexed environment.

Taken together, these findings suggest that Web of Science captures a relatively narrow segment of scholarly activity, primarily restricted to English-language publications. This further reinforces the role of database selectivity in shaping patterns of academic visibility. The observed concentration of citations is consistent with patterns identified across the other databases, although the degree of citation inequality varies across bibliometric systems.

### Faculty of Law, “Iustinianus Primus,” UKIM (Google Scholar Analysis)

The analysis of Google Scholar data for the Faculty of Law “Iustinianus Primus” at Ss. Cyril and Methodius University in Skopje (UKIM) includes a total of 69 academic staff members. Of these, 37 researchers (53.6%) maintain active Google Scholar profiles, while 32 (46.4%) are not represented in the database, indicating partial digital academic visibility and a fragmented presence within this widely used academic platform.

In terms of research output, a total of 1,665 publications were identified across the analyzed sample.

With regard to research impact, the total number of citations amounts to 3,244, corresponding to an average of 87.7 citations per researcher with an active profile. Citation distribution is highly uneven, with a small number of individuals accounting for a disproportionate share of total citations. The highest recorded value reaches 873 citations, while several researchers demonstrate minimal or zero citation impact.

This concentration becomes even more pronounced at the level of individual researchers. Notably, the most cited researcher alone accounts for 26.9% of all citations, while the top two researchers together account for 37.4% of total citations, clearly demonstrating the extent of citation concentration within the dataset. This pattern is consistent with cumulative advantage mechanisms observed in bibliometric research.

The analysis of citation distribution by language reveals a highly asymmetric pattern. Out of a total of 3,244 citations, English-language publications account for 3,009 citations (92.8%), overwhelmingly dominating academic visibility. In contrast, Macedonian-language publications account for only 90 citations (2.8%), while publications in other languages contribute 140 citations (4.3%), and Albanian-language outputs remain marginal with only five citations (0.15%).

To contextualize these citation patterns, it is useful to compare them with the distribution of publications by language. While English-language outputs dominate in terms of volume and generate a disproportionately high share of citations (92.8%), the pattern for non-English publications is more differentiated. Macedonian-language publications account for a relatively small share of citations, whereas the “Other languages” category comprises 106 publications generating 140 citations (approximately 1.32 citations per publication). This category, which includes primarily Serbian as well as Russian, German, Slovenian, and other languages, therefore represents a non-negligible component of UKIM's citation visibility.

Taken together, these findings indicate that citation impact is not proportionally distributed across languages, with English-language publications accounting for a disproportionately large share of citations within the analyzed dataset. Despite the predominance of English-language publications in overall citation volume, the results also indicate meaningful citation visibility for publications in other languages, suggesting that the observed language pattern cannot be reduced to a simple English-vs.-local-language distinction. Overall, the Google Scholar data for the Faculty of Law “Iustinianus Primus” reflects a dual structure. On the one hand, the presence of highly cited individual researchers indicates strong participation in international academic networks; on the other, the relatively limited profile coverage.

### Faculty of Law, “Iustinianus Primus,” UKIM (Scopus Coverage and Research Visibility)

The analysis of Scopus-indexed data reveals that out of a total of 69 academic staff members, 45 (65.2%) are represented in the Scopus database, while 24 (34.8%) are not indexed.

However, a more detailed examination indicates that profile presence does not necessarily correspond to accessible researcher-level citation data. Of the 45 identified Scopus author profiles, citation data were accessible for 27 researchers, while 18 identifiable profiles could not be opened or their researcher-level citation data could not be retrieved at the time of data collection. Among the 27 researchers with accessible citation data, 5 (18.5%) had zero recorded citations. Consequently, researcher-level citation data were available for 27 scholars, representing 39.1% of the total UKIM academic staff population.

In terms of research output, a total of 142 Scopus-indexed publications were identified across the analyzed sample, all published in English, reflecting strong alignment with international publication standards and indexing criteria.

With regard to research impact, the total number of citations amounts to 277, indicating a relatively modest level of measurable academic visibility. This is particularly notable when compared with the broader publication output observed in Google Scholar and illustrates the substantial differences in research visibility captured by the two databases. Citation patterns across individual researchers reveal a pronounced degree of concentration. The three most cited scholars account for approximately 61.7% of all citations, exceeding the combined citation output of the remaining researchers in the sample. Taken together, these findings suggest that while nominal inclusion in Scopus appears relatively high, effective research visibility—measured through indexed publications and citations—remains substantially lower. The discrepancy between profile presence and actual bibliometric performance highlights structural constraints within international indexing systems and raises important questions regarding the global dissemination of legal scholarship in the Macedonian academic context.

### Faculty of Law, “Iustinianus Primus,” UKIM (Web of Science Coverage and Research Activity)

The analysis of Web of Science (WoS) data shows that out of 69 academic staff members, 47 (68.1%) have publications indexed in the WoS database, while 22 (31.9%) are not represented.

In terms of research output, a total of 155 WoS-indexed publications were identified across the analyzed sample, generating 531 citations. This corresponds to approximately 3.43 citations per publication, indicating a moderate level of citation impact within the indexed dataset.

Citation patterns across individual researchers reveal a pronounced degree of concentration. A limited number of researchers account for a disproportionately large share of total citations, while a substantial portion of the sample records low or zero citation counts, indicating uneven research visibility within the WoS-indexed environment.

This concentration is particularly evident at the top of the distribution, where the three most cited researchers account for approximately 66.5% of total citations, demonstrating that overall citation performance is heavily driven by a very small number of individuals.

Taken together, these findings suggest that although Web of Science provides a more selective and output-based representation of research activity, citation impact remains unevenly distributed and concentrated among a limited number of highly visible individuals. This pattern further demonstrates that citation inequality is also present in Web of Science and that the degree of citation concentration varies across bibliometric databases.

### Faculty of Law, South East European University (Google Scholar Analysis)

The analysis of Google Scholar data for the Faculty of Law at South East European University (SEEU) includes a total of 21 academic staff members. Of these, 13 researchers (61.9%) maintain active Google Scholar profiles, while 8 (38.1%) are not represented in the database, indicating partial digital academic visibility within the institution.

In terms of research output, 541 publications were identified across the analyzed sample.

With regard to research impact, the total number of citations amounts to 922, corresponding to an average of 70.9 citations per researcher with an active profile. Citation distribution is highly uneven. A single researcher reaches 430 citations, while many others exhibit very low citation counts (as low as 3–4), and the majority remain below 50 citations, revealing a highly unequal distribution and a strong concentration of citation visibility. Notably, this single researcher alone accounts for approximately 46.6% of all citations, indicating a pronounced concentration of citation visibility within the dataset. This pattern is consistent with cumulative advantage mechanisms observed in bibliometric research.

In terms of language distribution, the dataset reveals a distinct structure. Out of 541 identified publications, English-language outputs dominate with 448 publications (82.8%), followed by Albanian with 80 publications (14.8%). Macedonian-language publications are minimal, accounting for only 6 publications (1.1%), while other languages represent 7 publications (1.3%).

The analysis of citation distribution by language reveals a similarly asymmetric pattern. English-language publications overwhelmingly dominate, accounting for 854 citations (92.6%). In contrast, Albanian-language publications account for 66 citations (7.2%), while Macedonian-language outputs contribute only 2 citations (0.2%), indicating extremely limited visibility.

When compared with the distribution of publications by language, this asymmetry becomes even more pronounced. While English-language outputs account for 448 publications, they generate 854 citations (92.6%), demonstrating substantially higher citation visibility within the analyzed dataset. Albanian-language publications, despite representing a notable share of total output (80 publications), account for only 66 citations (7.2%), while Macedonian-language publications (6 in total) contribute only 2 citations (0.2%).

Taken together, these findings indicate that citation impact is not proportionally distributed across languages, with English-language publications accounting for a disproportionately large share of citations within the analyzed dataset. While SEEU reflects a more linguistically diversified academic environment, academic visibility remains overwhelmingly concentrated in English-language publications, while outputs in Albanian and Macedonian demonstrate substantially lower citation visibility within the analyzed dataset.

### Faculty of Law, South East European University (Scopus Coverage and Research Visibility)

The analysis of Scopus-indexed data for the Faculty of Law at South East European University (SEEU) shows that out of 21 academic staff members, 15 (71.4%) are represented in the Scopus database, while 6 (28.6%) are not indexed, indicating partial coverage of academic staff within this indexing system. Researcher-level citation data were accessible for 14 of these 15 identifiable profiles; for one profile, the required researcher-level citation data could not be reliably retrieved during the data-collection period.

In terms of research output, a total of 86 Scopus-indexed publications were identified, reflecting a relatively limited volume of internationally visible research when compared to the broader publication activity captured by Google Scholar.

With regard to research impact, the total number of citations amounts to 150, indicating a substantial decrease compared to Google Scholar data. This difference is consistent with the more selective coverage and indexing scope of Scopus compared with Google Scholar. Citation patterns across individual researchers reveal a pronounced degree of concentration. A single researcher accounts for 118 citations (78.7% of the total), demonstrating a pronounced concentration of citations within the dataset. Taken together, these findings indicate that while SEEU demonstrates broader visibility in inclusive databases such as Google Scholar, its presence in Scopus remains substantially more limited. This disparity illustrates the different levels of research visibility captured by inclusive and selective bibliometric databases.

### Faculty of Law, South East European University (Web of Science Coverage and Research Activity)

The analysis of Web of Science (WoS) data for the Faculty of Law at South East European University (SEEU) indicates a relatively high level of database coverage. Out of 21 academic staff members, 18 (85.7%) have publications indexed in the WoS database, while 3 (14.3%) are not represented.

In terms of research output, a total of 92 publications are indexed in WoS, slightly exceeding the number recorded in Scopus. This suggests that, in the case of SEEU, WoS provides a somewhat broader representation of internationally indexed research output.

Regarding research impact, the total number of citations amounts to 229, representing an increase compared to Scopus but remaining substantially lower than Google Scholar. These differences should be interpreted in light of the distinct coverage and indexing characteristics of Web of Science, Scopus, and Google Scholar. Citation patterns across individual researchers reveal a pronounced degree of concentration. A single researcher accounts for 136 citations (59.4% of the total), demonstrating a pronounced concentration of citations within the dataset. Taken together, these findings indicate that SEEU demonstrates relatively strong coverage within Web of Science compared to Scopus, although overall citation impact remains modest. This further highlights structural differences between bibliometric databases and underscores the importance of database selection in evaluating academic performance.

### Citation inequality across institutions and databases

To provide a systematic assessment of citation inequality across institutions and databases, Table 2 presents the median citation count, interquartile range (IQR), proportion of researchers with zero citations, and Gini coefficient for each institutional and database combination.

The distributional indicators reveal substantial citation inequality across institutions and databases. Point estimates of the Gini coefficient range from 0.563 to 0.851, with relatively high levels of citation concentration observed in several Scopus and Web of Science distributions. However, the bootstrap confidence intervals indicate substantial uncertainty around some of these estimates, particularly where the number of researchers with accessible citation data is small. For example, the Gini coefficient for SEEU is 0.807 (95% CI: 0.306–0.859) in Scopus and 0.851 (95% CI: 0.607–0.896) in Web of Science, with considerable overlap between the confidence intervals. Accordingly, small differences between Gini point estimates should not be interpreted as evidence of substantively different levels of citation inequality. Median citation counts are generally lower in Scopus and Web of Science than in Google Scholar, while the proportion of researchers with zero citations is particularly high in Web of Science, reaching 57.1% for UGD, 34.0% for UKIM, and 33.3% for SEEU. Taken together, the distributional indicators demonstrate uneven citation visibility across researchers, while the bootstrap results emphasize the need for caution when comparing the precise magnitude of inequality across institution–database combinations with small *N*.

Sensitivity analysis: full-population citation inequality.

Because researcher coverage differs substantially across databases, particularly for Google Scholar at UKIM and SEEU, a sensitivity analysis was conducted using the complete staff population of each faculty. Researchers without an observable citation count in a database were coded as zero for this analysis. The results are presented in Table 3.

**Table 3:** Full-population sensitivity analysis of citation inequality.

Faculty	Database	*N*	Median citations	IQR (Q1–Q3)	Zero/unobservable *n* (%)	Gini
UGD	Google Scholar	21	20	10–66	0 (0.0%)	0.563
UGD	Scopus	21	0	0–7	11 (52.4%)	0.714
UGD	Web of Science	21	0	0–4	15 (71.4%)	0.830
UKIM	Google Scholar	69	3	0–39	33 (47.8%)	0.818
UKIM	Scopus	69	0	0–2	47 (68.1%)	0.888
UKIM	Web of Science	69	0	0–4	38 (55.1%)	0.891
SEEU	Google Scholar	21	13	0–45	8 (38.1%)	0.737
SEEU	Scopus	21	0	0–3	11 (52.4%)	0.871
SEEU	Web of Science	21	1	0–2	9 (42.9%)	0.872

Researchers without a profile or accessible citation count in a database were coded as having zero bibliometrically observable citations for this sensitivity analysis. These values indicate database-level non-observability and should not be interpreted as evidence of zero citations in an absolute sense.

The full-population sensitivity analysis confirms that differences in database coverage affect the magnitude of the inequality estimates. This effect is particularly visible for Google Scholar at UKIM, where the Gini coefficient increases from 0.660 in the profile-based analysis to 0.818 when all staff are included, and at SEEU, where it increases from 0.575 to 0.737. Under the full-population specification, Gini coefficients remain high in Scopus and Web of Science, although the differences between these two databases are very small at UKIM and SEEU. The sensitivity analysis therefore indicates that differential researcher coverage affects the magnitude of observed inequality and reinforces the need for caution when interpreting relatively small differences between database-specific Gini estimates.

### Comparative insight across databases

Taken together, the results across Google Scholar, Scopus, and Web of Science reveal substantial variation in academic visibility across databases. Google Scholar generally captures a broader volume of publications and citations, while Scopus and Web of Science provide more selective representations of scholarly output. However, the relative coverage of Scopus and Web of Science varies across institutions, indicating that database coverage does not follow a uniform hierarchical pattern. This comparison confirms that academic visibility is not a fixed attribute, but a database-dependent phenomenon. Each database captures a different segment of scholarly activity, shaped by its inclusion criteria and indexing scope. As a result, variations in citation counts and researcher representation across databases should not be interpreted solely as differences in academic performance, but as reflections of the structural characteristics of bibliometric systems.

A consistent pattern across all three databases is the uneven distribution of visibility. Citation impact is not evenly shared among researchers, but is concentrated among a limited number of highly cited individuals. The degree of concentration varies across institutions and databases, as confirmed by the distributional indicators reported in Table 2. Such a pattern is consistent with cumulative advantage dynamics described in the literature. At the same time, an association emerges between publication language and academic visibility. English-language publications account for the largest share of citation impact across the analyzed institutions, but the results also reveal a more differentiated pattern among non-English outputs. At UKIM, for example, publications classified under “Other languages” — primarily Serbian, as well as Russian, German, Slovenian, and other languages — account for 106 publications and 140 citations, indicating that non-English publication does not uniformly correspond to marginal citation visibility. The findings therefore suggest that the observed differences cannot be reduced to a simple English-vs.-local-language divide. Rather, citation visibility appears to vary across different publication languages and institutional contexts. These results indicate an association between publication language and citation visibility within the analyzed databases, without establishing a causal relationship between language choice and citation impact.

Institutional differences were also observed. UKIM recorded the highest overall citation volume, while UGD and SEEU displayed distinct patterns of publication output, language distribution, and citation concentration.

## Discussion

As shown in Table 1, academic visibility varies substantially depending on both database selection and institutional characteristics, confirming the layered and structurally conditioned nature of bibliometric representation. The findings support the argument that academic visibility is not a neutral reflection of research productivity, but a structurally conditioned outcome shaped by the interaction of database selectivity, language hierarchies, and cumulative citation dynamics.

First, the comparison across Google Scholar, Scopus, and Web of Science confirms that academic visibility is inherently database-dependent. Differences in researcher coverage, publication volume, and citation counts across the three databases reflect their distinct indexing criteria and coverage patterns and should not be interpreted as direct indicators of differences in research quality. In this sense, visibility is not merely measured by databases, but actively constructed through them.

Second, the results reveal a consistent association between publication language and citation visibility. Across the analyzed institutions and databases, English-language publications account for a disproportionately large share of citations relative to their share of total publication output. This pattern is consistent with broader language-related inequalities in scholarly communication, although the descriptive cross-sectional design of the study does not establish a causal relationship between publication language and citation impact. Publications in Macedonian and Albanian, while important for domestic legal scholarship, show substantially lower citation visibility in the analyzed databases, illustrating a tension between local relevance and international visibility. Third, the analysis reveals substantial inequality in the distribution of citation impact, consistent with cumulative advantage mechanisms described in the literature. Gini point estimates range from 0.563 to 0.851 across institutions and databases, indicating considerable citation concentration in several institution–database combinations. However, the bootstrap confidence intervals are relatively wide for some estimates, particularly where the number of researchers with accessible citation data is small. The intervals also overlap substantially in several cross-database comparisons. Consequently, the precise differences between individual Gini coefficients should be interpreted cautiously rather than as evidence of statistically distinct levels of citation inequality. Taken together with the median citation counts, interquartile ranges, and zero-citation proportions, the results nevertheless demonstrate that citation visibility is unevenly distributed across researchers, while the magnitude of this inequality is estimated with varying degrees of precision across institutions and databases. In the profile-based analysis, citation inequality is particularly pronounced in Web of Science, where the Gini coefficients reach 0.840 for UKIM and 0.851 for SEEU, while zero-citation proportions are also comparatively high. Median citation counts and interquartile ranges further demonstrate that citation visibility is unevenly distributed across researchers. Taken together, these indicators show that the degree of citation concentration varies across institutions and bibliometric databases.

The full-population sensitivity analysis further qualifies these findings. When researchers without an observable citation count are retained in the institutional denominator and coded as zero bibliometrically observable citations, inequality increases substantially in Google Scholar, particularly at UKIM (Gini = 0.818) and SEEU (Gini = 0.737). This indicates that profile-based analyses may understate citation inequality when database coverage is incomplete. Under the full-population specification, citation concentration remains high in both Scopus and Web of Science. However, the differences between these two databases are minimal at UKIM (0.888 and 0.891, respectively) and SEEU (0.871 and 0.872, respectively), and these small numerical differences should not be interpreted as substantively distinct levels of inequality. Thus, differences in database coverage affect the magnitude of the observed inequalities and should be taken into account when interpreting cross-database differences.

Taken together, the findings suggest that academic visibility operates as a layered system in which different databases capture different segments of scholarly activity according to their respective inclusion criteria. From a theoretical perspective, the study contributes to ongoing debates on inequality in global knowledge production by demonstrating that structural asymmetries are not limited to differences between countries or institutions, but are also reproduced within small academic systems Even within a single national context, academic visibility remains highly stratified, with substantial disparities between researchers in terms of access to international recognition.

The observed differences across databases also reflect differences in indexing architecture and coverage rather than inherent differences in research quality. Google Scholar captures a broader range of publication types, including local-language outputs and non-indexed journals, whereas Scopus and Web of Science apply more selective indexing criteria focused primarily on internationally recognized journals. Consequently, lower citation counts in the latter databases should not be interpreted as evidence of lower scholarly quality, but should be considered in light of their more restrictive coverage and indexing practices. These limitations further reinforce the argument that academic visibility should not be treated as a purely objective measure of research performance, but as a product of both structural and technical conditions.

The findings also carry important implications for research evaluation and institutional policy. Reliance on a single bibliometric source—particularly highly selective databases—may lead to partial or distorted assessments of academic performance, especially in disciplines such as law, where local-language publications remain central. At the same time, the dominance of English-language outputs in citation metrics may incentivize publication strategies that prioritize international visibility over local relevance. This incentive is also reinforced by the national research-policy framework in Macedonia. Although the current Law on Higher Education requires publications in “reference scientific publications” for academic promotion, this category is defined broadly and does not establish publication specifically in Web of Science or Scopus as an exclusive general requirement (Law on Higher Education, 2018; as amended). At the same time, the national framework provides direct financial incentives for publication in journals indexed in these databases. The Ministry of Education and Science provides reimbursement of publication costs and one-time financial awards for eligible publications in journals indexed in Web of Science or Scopus (Ministry of Education Science, 2026a,b). Similar incentives also operate at the institutional level. At UGD, for example, eligibility for annual awards and recognition for scientific activity explicitly includes publication in journals indexed in Scopus or Web of Science (Goce Delcev University, 2025). Consequently, visibility in these databases is not merely a descriptive bibliometric outcome but may also carry direct material and institutional value for researchers. This policy orientation may become even more consequential if the proposed new Law on Higher Education is adopted, as the proposal introduces substantially stricter publication requirements for academic promotion, including a specified number of publications in journals indexed in Web of Science or Scopus (Ministry of Education Science, 2026c).

## Conclusion

This study examined academic visibility within legal scholarship in a small-state context through a comparative analysis of Google Scholar, Scopus, and Web of Science data from three leading law faculties in North Macedonia: Goce Delcev University (UGD), Ss. Cyril and Methodius University in Skopje (UKIM), and South East European University (SEEU).

The findings show that academic visibility varies substantially across Google Scholar, Scopus, and Web of Science, reflecting differences in database coverage, indexing practices, and measurable research visibility rather than a uniform representation of scholarly activity. Publication language emerges as an important factor associated with differences in citation visibility. English-language publications account for the largest share of citations across the analyzed institutions, while citation patterns among non-English outputs are more differentiated. Although Macedonian- and Albanian-language publications generally show lower citation visibility than English-language outputs, the UKIM data demonstrate that publications in other languages can also achieve meaningful citation visibility. The findings therefore point to a more complex relationship between publication language and academic visibility than a simple English-vs.-local-language divide. However, citation patterns among non-English publications are not uniform. At UKIM, publications classified as “Other languages” account for 106 publications and 140 citations, indicating that non-English publication does not uniformly correspond to marginal citation visibility. The study also reveals substantial inequality in the distribution of citation impact. This pattern is supported by the distributional indicators, including median citation counts, interquartile ranges (IQR), zero-citation proportions, and Gini coefficients, which demonstrate consistently uneven citation distributions across institutions and databases. Bootstrap confidence intervals indicate, however, that the precision of some Gini estimates is limited, particularly in institution–database combinations with small numbers of researchers with accessible citation data. Accordingly, relatively small differences between individual Gini coefficients should be interpreted cautiously. The full-population sensitivity analysis further shows that incomplete database coverage can substantially affect the magnitude of estimated citation inequality, particularly in Google Scholar. Nevertheless, citation concentration remains evident across the analyzed bibliometric systems. The findings therefore demonstrate that differences in researcher coverage partly shape measured inequality, while the precise magnitude of cross-database differences remains subject to uncertainty. These findings indicate that academic visibility is unevenly distributed across researchers and that the degree of concentration varies across bibliometric systems. Overall, the study demonstrates that bibliometric indicators should be interpreted within the context of database selectivity, language, and disciplinary publication practices. Rather than providing a single objective measure of research performance, different databases construct different representations of academic visibility. These findings contribute to ongoing debates on responsible research assessment and highlight the need for context-sensitive approaches to evaluating legal scholarship in small-state academic systems. More balanced and context-sensitive approaches to research evaluation are therefore needed, especially for smaller academic systems operating outside the major centers of global knowledge production. Recognizing the role of database inclusion, language hierarchies, and citation inequality is essential for developing a more nuanced understanding of academic visibility and scholarly impact in diverse publishing environments.

## Limitations and future research

This study is subject to several limitations that should be considered when interpreting the findings. First, the analysis is based on a cross-sectional dataset, reflecting the state of academic profiles at a specific point in time. As bibliometric data are continuously updated, the results may evolve as new publications and citations are added.

Second, differences between databases in terms of coverage, indexing criteria, and data structure limit the direct comparability of results. While Google Scholar provides broad and inclusive coverage, Scopus and Web of Science apply more selective criteria, affecting both the number of indexed publications and citation counts.

Third, although the institutional populations differ in their academic-rank composition, the study does not model academic rank or career age as individual-level determinants of citation performance. Academic rank is reported descriptively to provide context for cross-institutional comparisons, but career age was not independently measured. The year of first indexed publication was not treated as a proxy for career age because it reflects database-specific coverage and may not correspond to the actual beginning of an academic career. Consequently, differences in citation volume and citation inequality across institutions may partly reflect differences in staff seniority and career stage, and the cross-institutional comparisons should be interpreted with this limitation in mind.

Fifth, the study focuses on a single academic field within a specific national context, which may limit the generalizability of the findings to other disciplines or academic systems.

Future research could build on these findings in several important ways. A longitudinal approach would provide deeper insight into how academic visibility evolves over time, particularly in relation to changes in publication strategies and database indexing practices. Comparative analyses across different disciplines could help determine whether the observed patterns—especially those related to language dominance and citation inequality—are specific to legal scholarship or reflect broader trends within the social sciences and humanities.

Future research could extend this comparative bibliometric approach by incorporating researcher-level demographic and professional characteristics, such as career stage, language background, workload, and institutional responsibilities, in order to examine potential individual-level determinants of bibliometric visibility.

In addition, future studies could examine the role of institutional policies, funding structures, and international collaborations in shaping publication behavior and database visibility. Finally, qualitative approaches could complement bibliometric findings by exploring how researchers perceive academic visibility, language choice, and publication strategies across different contexts.

## Data Availability

The original contributions presented in the study are included in the article/Supplementary material, further inquiries can be directed to the corresponding author.
